# Surgery of the Accessory Sinuses1Read before the Buffalo Academy of Medicine, November 6, 1894, and Roswell Park Medical Club.

**Published:** 1895-01

**Authors:** Geo. F. Cott

**Affiliations:** Instructor in Laryngology at the University Dispensary


Buffalo Medical and Surgical Journal

Vol. XXXIV. JANUARY, 1895. No. 6.

### Original Communications.

### SURGERY OF THE ACCESSORY SINUSES.[1]

By GEO. F. COTT, M. D.,
Instructor in Laryngology at the University Dispensary.

The surgery of the accessory sinuses has largely occupied the attention lately of the American and British laryngological associations. In discussing the subject many opinions were advanced regarding the symptomatology and treatment. Since the subject is of great importance to the practitioner who has very little time to follow up all diseases treated mainly by specialists, but which are necessary for him to recognize, I concluded to formulate the principal symptoms, whereby, as near as is possible, to differentiate between the affections of the different sinuses and to give the treatment which has yielded the best results. Cases of sinusitis, primary or secondary, are often and easily mistaken for ozena, which is reasonable and excusable when it occurs as an acute disease associated with, or following an acute affection of the nasal chambers; when, however, it is chronic, in which condition the physician usually meets with it, a proper diagnosis of sinusitis can be made if certain symptoms and tests are observed. It must be admitted, however, that a positive diagnosis as to the particular sinus affected is often difficult to determine. To make it as easy as possible etiology and pathology will not be treated of in this paper, leaving the mind of the busy practitioner unhampered and still make the desired impression, which is, to recognize the affection whenever the opportunity presents itself.

The accessory sinuses are: Frontal, anterior and posterior ethmoidal and sphenoidal cells, and the antra of Highmore or maxillary sinuses. The posterior ethmoid and sphenoid cells open into the superior meati by one or more openings, the frontal sinus,

1. Read before the Buffalo Academy of Medicine, November 6, 1894, and Roswell Park Medical Club.

the anterior ethmoid cells and the antra of Highmore into the middle meati, the nasal or lachrymal duct, open into the inferior meati.

### DISEASES OF FRONTAL SINUS.

When the patient comes under observation he will complain of matter discharging from the nose; if that is pent up he will have frontal headache, the severity of which depends upon the amount of pressure exerted by the retained fluid. If relief is not obtained the roof of the orbit will be crowded downward and displacement of the eyeball is produced with diplopia and amaurosis; or, pressure may be posteriorly, in which case pressure upon the brain will cause symptoms referable to that organ. If the pus escapes into the nose the above symptoms will be absent excepting the headache, which is more or less constant. Pus discharges continually and is in appearance bright yellow. An examination at this stage will show pus covering the inferior turbinate; to ascertain its source it should be wiped off with spray and swab, then the parts cocainized, which causes the tissue to contract when a little pus will be seen to emerge from the infundibular opening in the middle meatus. The above symptoms taken together will point to trouble in the frontal sinus.

### TREATMENT.

In acute catarrhal affections of the frontal sinus, ordinary treatment applied to the nasal cavities causes the symptoms rapidly to disappear, providing the opening in the middle meatus is patulous. When chronic, exit for the pus must be established, which can be done by removing the hypertrophied tissue over the middle turbinated bone; but, if this is not sufficient, the anterior extremity of the middle turbinated bone may also be removed and the nasal cavity kept as nearly aseptic as possible. If further interference becomes necessary, an opening can be made where the pressure seems to be greatest, over the inner angle of the eye. The opening should be large enough to explore the sinus with the little finger, and removing granulations, carious bone, polypi, etc., with the sharp spoon, passing a probe through the infundibulum into the middle meatus, to be sure it is open, then wash it out and pack with antiseptic gauze; dressing to be repeated daily for several weeks. In the meantime, the nasal cavity is to be kept scrupulously clean.

### SPHENOIDAL SINUS.

In disease of the sphenoidal sinus, pus of a bright yellow color will usually be noticed, dropping down the pharynx by reason of the opening situated immediately above and a little posterior to the middle turbinated bone; deep-seated pain referable to the side affected, sometimes very severe, involving all parts of distribution of the fifth nerve. Owing to the close proximity of the sinus to the optic foramin, pressure upon the optic nerve causes impairment of vision or total blindness, which may come on suddenly. If the condition is not relieved, the disease affects the bony structure, and the character of the pus changes often to the caseous variety. If pus cannot escape, it is liable, by pressure, to cause ptosis, strabismus and exophthalmus. When necrosis occurs there is danger of extension of the inflammation to the brain, or, as has occurred in one case, extending from the sphenoidal to the cavernous sinus, causing destruction of bone and hemorrhage from mouth and nose, and finally death.

### TREATMENT.

Any obstructing substance must be removed, such as polypi or hypertrophies, and if any nasal inflammation be present, it must be reduced by the usual mild and soothing methods. A probe can then be passed in search of denuded or carious bone, which can be scraped out with a sharp spoon or a trephine, propelled by the electric motor or surgical engine, always using the posterior rhinoscopic mirror, as the operation is extremely dangerous by easily penetrating the roof and entering the brain. After opening the sinus careful search must be made for dead bone or caseous material, cleaned out with the spoon or curette and washed with antiseptic lotions.

### ETHMOIDAL DISEASE.

Acute disease of these cells, caused by extension from the nasal cavity, usually subsides when the latter is relieved. It may, however, become chronic, and through closure of their openings retain the secretion which degenerates and then causes the following symptoms: pain referréd to the root of the nose and back of the eye; if pus escapes the fetor is not so marked to the patient, as the sense of smell is often impaired by the discharge preventing the odoriferous particles from coming in contact with the olfactory filaments. The lamella in this region being very thin, are easily broken down and appear in the pus as small gritty particles. When the cells are distended the bone gives way on the side of least resistance, which is toward the orbit, crowding the eyeball forward and outward and impairing vision. A swelling is then observed at the inner angle of the orbit, when crepitation may be produced by pressure, supposedly due to emphysema. I met a case some years ago, in which the eye was removed and a large amount of pus escaped. The patient died, however, from other causes, which prevented me from gathering further data.

### TREATMENT.

The nasal cavities should be thoroughly cleansed, then the anterior part of the middle turbinated bone removed and the opening brought into view. This is all that is necessary, as the anterior cells often extend into the turbinated bone, and pus freely escapes. A sharp spoon may be passed into the cavity and it scraped out. The nose may be slit up, to gain easier access, which otherwise is extremely difficult. The cavity is then washed out and the nose packed with wool or gauze. This, as well as sphenoidal disease, is an extremely grave affection and requires considerable dexterity in operating.

### DISEASE OF THE ANTRUM.

In disease of the antrum of Highmore, or maxillary sinus, the diagnosis is not so difficult, especially when uncomplicated. If, however, the affection is overlooked there is usually no danger to the patient, unless necrosis takes place.

The symptoms present, especially when the osteum maxillaræ is occluded, are : Pain in the affected region, with a sense of fullness and weight below the orbit; if obstruction lasts very long, pain will be extreme, extending over the whole side of the face. Mastication may become difficult, the teeth appearing longer than normal. After the pus has been evacuated these symptoms disappear and a more or less intermittent discharge takes place. When the disease is chronic, in which condition it is usually found, because it is often secondary, there will be very little pain, some swelling may be noticed over the nasal process of the superior maxillary bone. The patient complains of pus running down the throat at night, but is discharged or blown out of the nose during the daytime, but not immediately after rising in the morning. In disease of the other sinuses, position will make no difference.

A modern method of diagnosis is by transillumination. By placing an electric lamp in the mouth and closing the lips over it, having the room dark, then turning on the current, one side will be illuminated and the other containing pus will remain dark. When the two cavities are affected both will remain dark. This is only possible, however, when the antrum is pretty well filled with opaque fluid or other substance. If a cyst be present illumination will be complete, but other symptoms, such as bulging, etc., will be noticed, which may be and has been mistaken for sarcoma (votalini). In case of a small amount of pus the above method will avail nothing. Therefore Davidsohn has made the positive statement that transillumination of the *eye* is the only certain sign of abscess of the antrum. Illumination of the eye is always a sign of a healthy antrum; a dark eye, however, is not a positive sign of disease in this cavity. Illumination may be prevented by undue narrowing of the antral cavity, asymmetry of the hard palate or an excessively arched palate, preventing the rays of light from passing through the antral cavity. Some stress might be laid upon absence of luminous impression on one side by the patient himself. With a lamp in the mouth of a healthy subject whose eyes are closed, a luminous impression is produced upon the lower part of the retina. When empyema is present this impression is not observed. A very simple method giving most positive knowledge is exploratory puncture in the lower meatus and injecting dil. $H_2O_2$ the ozonizing process speedily becomes manifest in the natural opening in the middle meatus.

Differential diagnosis is often very difficult, but if you will bear in mind that frontal (suppurative) disease is rare, and sphenoidal disease extremely grave, you will have safer ground to work on. I believe ethmoidal and sphenoidal affections occur more often than is observed. Many cases of death from meningitis might be explained if the history regarding nasal symptoms and pain before the meningeal attack occurred were ascertained. To differentiate between pyosinusitis and other affections of the nasal cavities is not so difficult as it may seem. The sources of pus discharged from the nose are: purulent rhinitis in children, syphilis, rhinolith or other foreign body, neoplasm, nasal diphtheria and the several diseased sinuses. Neoplasm, sinusitis, syphilis and foreign bodies usually cause unilateral discharge, but the accompanying symptoms of each make the differential diagnosis easy. Pus from the sinuses is uniformly bright yellow, unless necrosis supervene. When pent up for any length of time it has the odor of sulphuretted hydrogen. Very little odor is observed if the flow is unobstructed. Although recognition of sinusitis is an easy matter it is sometimes very difficult to locate the source of pus. By studying the locations of the several openings in the nasal cavities we find pus discharging from the frontal sinus, anterior ethmoid cells and the antrum into the middle meatus and out of the nostrils, while that of the posterior ethmoid and sphenoid cells passes down the pharynx since these latter openings are situated in the superior meatus just above the posterior extremity of the middle turbinated body. Discharge from the antrum is usually intermittent, more abundant in the morning, the flow being favored by reclining the head, while the upright position favors the flow from the other cavities, the discharge being continuous. Pain is unilateral and referred to the affected sinus. Exophthalmus is the rule in ethmoid disease and may be present in sphenoidal, rare with antral unless the abscess ruptures into the orbit. Bulging of the orbital plate does occasionally occur in frontal disease, but is the rule in ethmoidal, differing in kind however, being forced downward and outward, the eyeball not protruding so much. Sudden blindness occurs, probably, only in disease of the sphenoidal sinuses, by pressing upon the optic nerve as it passes through the optic foramen. Ptosis is produced by pressure on the fifth nerve in its passage through the sphenoidal fissure, likewise strabismus. The frequency with which the different sinuses become affected is also of diagnostic value; that of the antrum is more common, ethmoid and sphenoidal are less frequent, and the rarest of all is frontal disease.

### TREATMENT OF ANTRUM.

I will not burden you with a description of all the methods of treatment now in vogue, but will mention those which will appeal to you as most rational. Puncturing through the inferior meatus with knife or drill may be readily accomplished and the cavity washed out daily. The patient cannot manipulate the syringe very well, however, because of the pain produced in its introduction, and cocainizing becomes necessary. A better method is opening through the alveolus, especially where there is a tooth missing, using the electric motor, surgical engine, or a hand drill of three-sixteenths-inch diameter. A metal tube may then be inserted and the patient be taught the use of a syringe for daily flushings. As antral disease is often due to carious teeth, this method of operating seems most rational. If upon exploration the cavity is found denuded, it becomes necessary to pack it with gauze; for this purpose an opening should be made with drill and chisel back of the canine fossa, drilling backward, upward and outward, making an opening large enough to explore the cavity with the little finger for dead bone, foreign body, such as a supernumerous tooth, bony partitions, etc., then flushed with diluted $H_2O_2$ followed by a weak sublimate solution or boracic water, then packed with gauze daily. When no denuded bone can be detected, a counter-opening may be made into the inferior meatus, behind the opening into this meatus of the lachrymal or nasal duct, and the patient taught to flush the cavity two or three times daily by first rinsing the mouth thoroughly, then taking a large draught of 5 per cent. boroglyceride, diluted $H_2O_2$ or boracic solution, and forcing it under the lip into the antrum, when it will emerge from the counter-opening in the inferior meatus. This method, it is claimed, will cure the trouble in one or two months. The writer has given a fair trial to all these operations and to numerous drugs to facilitate the process of healing. Iodoform in powder and solution of 2 per cent. alboline, sub-iodide of bismuth and aristol in the same way, sublimate solution 1 to 1,000 and 1 to 2,000, saturated solution of boracic acid, 2 per cent. solution of potassium permanganate, fifteen vol. solution of $H_2O_2$ full strength and diluted, also pyrozone, which is a 25 per cent. solution of $H_2O_2$ and is probably the best pus finder we have. I now confine the flushings to diluted $H O_2$ and 5 per cent. boroglyceride, the object being cleanliness and to prevent the accumulation of pus. The duration of the disease is from one month to several years. The particular kind of operation selected is entirely governed by conditions present.

In conclusion I will briefly cite two extreme cases:

A woman, aged 30, referred by Dr. Eugene E. Storck, complained of pus running down her throat at night and blowing out a large quantity during the day-time, usually soiling ten handkerchiefs a day, and some swelling and pain in left cheek. She was very much emaciated. After preliminary treatment an opening was made over the first bicuspid tooth and the cavity explored. It was found entirely denuded. After using the sharp spoon for possible granulations, the cavity was flushed and packed. After several months of this treatment the opening was allowed to close, as there was no more pus escaping. This patient suffered over a year before coming under treatment, which extended over a period of six months. There is still more discharge from that nostril than from the other, but its character is not that of pus, according to the patient's statement, nor is there any more discharge down the pharynx at night.

The second case was that of a man aged 40, recently referred by Dr. J. C. Thompson, in which there was a great discharge of pus of four weeks' standing. The first molar was missing, an opening was made through that socket with a drill and the antrum washed out daily. No other treatment was used. The patient recovered in three weeks.

BIBLIOGRAPHY.—Brown, M. R., *Medical Record*, April 1, 1893; Wolfenstein, Julius, *New York Medical Journal*, August 5, 1893; Caldwell, *New York Medical Record*, April 8, 1893; Wright, G. A., *Medical Chronicle*, July, 1894; Mr. Mayo Collier, British Laryngological Association, July 13, 1894; Dr. Dundas Grant, British Laryngological Association, July 13, 1894; Dr. Arthur Sandford, British Laryngological Association, July 13, 1894; Mr. Mayo Collier, *Lancet*, January 27, 1894; Schaeffer, Dr., *Deutsche Med. Woch.*, 1892, No. 47; Burger, Dr., *Review Mens. de Laryng.*, January 1, 1894; Codwell, G. W.; Dr. Sevens Spicer, British Medical Association, August, 1894; C. J. Symonds, M. S., F. R. C. S., England, British Medical Association, August, 1894; G. McDonald, M. D., British Medical Association, August, 1894; Lichtwitz, *Therap. Monats.*, 1893, No. 9; Oaks, *Medical News*, September 2, 1893; Major, Geo. W., *New York Medical Journal*, August 19, 1893; Diseases of Nose and Throat, Bosworth; "System," Nose, Throat and Ear, Burnett.

